# Comparative Long-Term Effect of Three Anti-P2Y12 Drugs after Percutaneous Angioplasty: An Observational Study Based on Electronic Drug Adherence Monitoring

**DOI:** 10.3389/fphar.2017.00738

**Published:** 2017-10-25

**Authors:** Valentina Forni Ogna, Isabelle Bassi, Isabelle Menetrey, Olivier Muller, Eric Tousset, Pierre Fontana, Eric Eeckhout, Chin B. Eap, Bernard Vrijens, Michel Burnier, Grégoire Wuerzner

**Affiliations:** ^1^Service of Nephrology and Hypertension, Department of Medicine, Lausanne University Hospital, Lausanne, Switzerland; ^2^Service of Cardiology, Lausanne University Hospital, Lausanne, Switzerland; ^3^Aardex Group, Visé, Belgium; ^4^Division of Angiology and Hemostasis, Geneva University Hospital, Geneva, Switzerland; ^5^Geneva Platelet Group of the Faculty of Medicine, University of Geneva, Geneva, Switzerland; ^6^Unit of Biochemistry and Clinical Psychopharmacology, Center for Psychiatric Neuroscience, Department of Psychiatry, Lausanne University Hospital, Lausanne, Switzerland; ^7^The Geneva-Lausanne School of Pharmacy (EPGL), University of Geneva, Geneva, Switzerland

**Keywords:** anti-P2Y12 drugs, VASP-PRI, elective coronary stenting, drug adherence, MEMS^®^, percutaneous coronary angioplasty intervention

## Abstract

**Aims:** Dual platelet inhibition using anti-P2Y12 drugs and aspirin is the standard of care in patients after percutaneous coronary interventions (PCI). Prasugrel and ticagrelor have been shown to be more potent than clopidogrel with less high on-treatment platelet reactivity. Whether differences in long-term adherence to these drugs can partly explain different antiplatelet efficacy has not been studied so far. The objective was to compare the long-term P2Y12 receptor inhibition and drug adherence to different anti-P2Y12 drugs, and to assess the impact of adherence on the pharmacodynamic effect.

**Methods:** Monocentric, prospective, observational study. Stable outpatients treated with clopidogrel 75 mg once daily, prasugrel 10 mg once daily or ticagrelor 90 mg twice daily after PCI with stent implantation were included. Drug adherence was recorded during 6 months using electronic monitoring. Platelet responsiveness was assessed with the vasodilator-stimulated phosphoprotein platelet reactivity index (VASP-PRI) at inclusion, 3 and 6 months.

**Results:** 120 patients had VASP-PRI and adherence data available. At 6-months, mean VASP-PRI (±SD) was 17.7 ± 11.0% with ticagrelor, 29.2 ± 15.5% with prasugrel and 47.2 ± 17.6% with clopidogrel (ANOVA, *P* < 0.0001).

Median [IQR] taking adherence was 96 [82–100]% with ticagrelor, 100 [97–101]% with prasugrel and 100 [99–101]% with clopidogrel (*p* = 0.0001). Median [IQR] correct dosing was 88 [73–95]% with ticagrelor, 97 [92.5–98]% with prasugrel and 98 [96–99]% with clopidogrel (*p* = 0.0001).

Anti-P2Y12 drug (*p* ≤ 0.001) and diabetes (*p* = 0.014) emerged as predictors of poor antiplatelet response after adjusting for age, BMI, sex, and CYP2C19^∗^2 carriers status.

**Conclusion:** Drug adherence to anti-P2Y12 drugs assessed with electronic monitoring was very high. However, anti-P2Y12 drugs showed significant differences in antiplatelet activity, with newer anti-P2Y12 drugs ticagrelor and prasugrel exerting a stronger P2Y12 receptor inhibition.

These data suggest that pharmacokinetic-pharmacodynamic differences between oral anti-P2Y12 drugs are more important than adherence in determining antiplatelet efficacy when adherence to prescription is high.

The study was registered (Current Controlled Trials ISRCTN85949729).

## Introduction

Dual antiplatelet therapy (DAPT) with aspirin and an oral anti-P2Y12 drug targeting the ADP-induced platelet activation is the recommended standard of care for secondary prevention after elective percutaneous coronary interventions (PCI) (Silber et al., 2005; Levine et al., 2011). Within the acute phase (1 month) after stent implantation, the main concern is early stent thrombosis due to DAPT interruption/non-adherence (Naidu et al., 2012). Recently non-adherence to DAPT in the subacute phase (1–6 months) of PCI with second generation drug eluted stents (DES) implantation has been correlated with the occurrence of thrombotic outcomes at 1 year (Cutlip et al., 2015).

Clopidogrel has been the oral anti-P2Y12 drug of choice for years, but has shown significant limitations, due to its pharmacokinetic and pharmacodynamic characteristics. It is a prodrug requiring transformation in the liver resulting in unpredictable and delayed onset of action, has modest, variable and irreversible antiplatelet efficacy, its action is influenced by several demographic and genetic determinants. (Hochholzer et al., 2010) The new anti-2Y12 drugs, prasugrel (Jernberg et al., 2006; Wiviott et al., 2007) and ticagrelor (Wallentin et al., 2008, 2009) have been shown to have more predictable and greater antiplatelet efficacy than clopidogrel. Nevertheless, the short plasma half-life of 7–8.5 h of ticagrelor mandate its twice daily dosing (b.i.d.) (Butler and Teng, 2010), which may lead to slightly more missed doses compared to the once daily (o.d.) regimen of clopidogrel and prasugrel (Comte et al., 2007). To our knowledge, data comparing the impact of adherence on the long-term (6–12 months after PCI) antiplatelet efficacy of different anti-P2Y12 drugs are not available so far.

The main objective of this study was therefore to compare the long-term anti-P2Y12 receptor inhibition and drug adherence profile of the three anti-P2Y12 drugs after elective PCI, and to investigate the impact of drug adherence on the long-term platelet inhibition.

## Materials and Methods

### Study Population and Design

We screened all consecutive adult patients admitted to the cardiology interventional unit of the Lausanne University Hospital for elective PCI from April 2010 through January 2014. Patients were enrolled if they had undergone PCI with implantation of at least one stent and had been prescribed maintenance treatment with an anti-P2Y12 drug (clopidogrel 75 mg o.d. or prasugrel 10 mg o.d. or ticagrelor 180 mg b.i.d.) for at least 6 months. The main exclusion criteria were: non-ST-segment elevation myocardial infarction or ST-segment elevation within 30 days prior to inclusion.

Eligible patients were randomly assigned (block size 1:2) to a follow-up without or with anti-P2Y12 drug adherence monitoring using the Medication Event Monitoring System (MEMS^®^).

For this observational analysis, we only selected patients of the active arm of the trial. These patients all had electronic monitoring of their anti-P2Y12 drug intake.

The vasodilator-stimulated-phosphoprotein (VASP) platelet-reactivity-index (PRI) was measured at inclusion, 3 and 6 months as marker of P2Y12 receptor antagonists’ efficacy. The primary endpoint of the study was the VASP-PRI at 6 months.

All patients gave their written informed consent. The study was approved by the local Ethics Committee of the University of Lausanne, Switzerland, and was carried out in accordance of the principles of the Declaration of Helsinki. The study was registered (Current Controlled Trials ISRCTN85949729).

### Study Procedures

#### Platelet Function Assay and Evaluation of Anti-P2Y12 Drug Efficacy

Platelets activity was assessed with citrated whole blood using the Platelet VASP/P2Y12 assay (VASP-assay, Platelet VASP/P2Y12, Biocytex, Marseille, France), a flow cytometry assay that specifically assesses the antiplatelet efficacy of anti-P2Y12 drugs as described previously (Aleil et al., 2005; Forni Ogna et al., 2016). Results are expressed in terms of platelet reactivity index (PRI). Poor response to the anti-P2Y12 drug (high on-treatment platelet response, HPR) was defined as a VASP-PRI ≥ 50%. This value has been shown to be the optimal cut-off to exclude major cardiovascular events after PCI (Tantry et al., 2013).

#### Genotyping

Genomic DNA was extracted from EDTA blood samples with the FlexiGene DNA extraction kit (QIAGEN, Basel, Switzerland). The following single nucleotide polymorphism (SNPs) were detected by real-time PCR with 5′-nuclease allelic discrimination assays (ABI PRISM 7000; Applied Biosystems, Luzern, Switzerland) (Jaquenoud Sirot et al., 2009): CYP2C19^∗^2, CYP2C19^∗^3, CYP2C19^∗^17. Patients were classified into 6 predicted phenotypes: carriers of two functional (^∗^1) alleles (^∗^1/^∗^1; extensive metabolizers), carriers of 1 functional allele and 1 non-functional (^∗^2) alleles (^∗^1/^∗^2; intermediate metabolizers), carriers of 1 gain-of-function allele (^∗^17) and 1 non-functional allele (^∗^17/^∗^2; intermediate metabolizers), carriers of only non-functional allele (^∗^2/^∗^2; poor metabolizers), carriers of only gain of function allele (^∗^17/^∗^17; rapid metabolizers) and carriers of 1 functional and 1 gain of function allele (^∗^1/^∗^17; rapid metabolizers). Subjects were also dichotomously classified as “carriers” of at least 1 non-functional allele (^∗^1/^∗^2^∗, ∗^17/^∗^2, and ^∗^2/^∗^2) and “non-carriers” (^∗^1/^∗^1, ^∗^1/^∗^17, and ^∗^17/^∗^17).

#### Adherence Monitoring

Adherence monitoring: medication adherence is the process by which patients take their medication as prescribed. It is composed of three elements: (1) initiation of therapy, (2) implementation of the dosing regimen, and, eventually, (3) discontinuation of therapy. In this study, all patients initiated therapy and dosing history data were censored at treatment discontinuation. Therefore, the adherence focus is the implementation of the dosing regimens. The Medication Event Monitoring System – MEMS^®^ was used to automatically compile medication dosing history data during the 6-months study period. This system entails the use of a tablet bottle closure that electronically records the time and date when the cap is removed. The data are collected, recorded and can be processed to generate a graphic representation of the dates and times of bottle openings.

Adherence data summarize daily implementation of the dosing regimens using two different summary statistics: (Wessels et al., 2012)

(1) *Taking adherence (Tac)*: the proportion of prescribed drug taken during the time window, calculated as:
N° of openings/N° of prescribed doses × 100.
The *Tac* measure reflects both the average dose received over a given period and the total dose received over that period. However, it fails to distinguish between a patient who takes his medication regularly and a patient who balances periods of underdosing with periods of overdosing and it captures no information about the precise timing of drug intake.
(2) *Correct dosing (Cod):* the percentage of days with correct number of doses taken, calculated as:
Number of days with N° of openings as prescribed/N° of monitored days × 100.
The *Cod* measure captures some measure of the closeness to ‘correct adherence.’ It reflects the degree of regularity in lifestyle.
In the adherence summary, we did not consider periods without drug intake because of medical prescription (for example after a bleeding episode).

### Safety

Bleeding complications were characterized according to the GUSTO (Global Utilization of Streptokinase and Tpa for Occluded Arteries) definition for bleeding (Mehran et al., 2011).

### Statistical Analysis

The VASP-PRI results were compared between groups using ANOVA; adherence results were compared between groups using the Kruskal–Wallis test.

Linear mixed-effects models were used to model longitudinal VASP-PRI and HPR data.

The analyses of the determinants of poor response to the anti-P2Y12 drug were performed in all treatment groups: The analyses of VASP-PRI determinants was performed in all treatment groups: in a logistic regressions for models adjusting for other covariates, we included the measures of HPR at 6 months as dependent variables and anti-P2Y12 drug treatment group, age, sex and CYP2C19^∗^2 carriers status as covariates. The analyses were performed using STATA 14.0 (Stata Corp, College Station, TX, United States). *P* < 0.05 was considered as statistically significant.

## Results

From April 2010 through January 2014, a total of 822 patients were screened and 191 recruited and randomized in two groups, with and without drug adherence monitoring. Only the 129 patients having their adherence of anti-P2Y12 drug monitored using the Medication Event Monitoring System (MEMS^®^) were included in this analysis. Supplementary Table 1 shows the demographic characteristics of included and not included patients, who only slightly differed in the “Indication to revascularisation” item.

**FIGURE 1:**
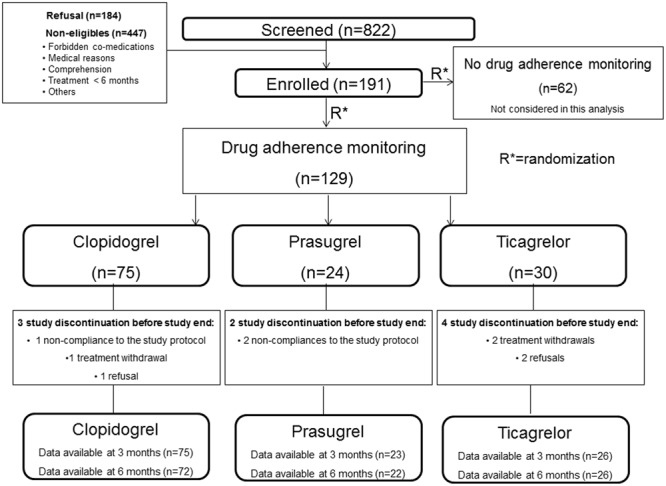
Flow diagram of the study.

Of the 129 patients included, 75 were treated with clopidogrel, 24 with prasugrel and 30 with ticagrelor. **Figure 1** shows the flow-chart of the study. **Tables 1, 2** show the demographic, clinical and biological characteristics of the patients at inclusion, according to the ongoing anti-P2Y12 drug. Patients with clopidogrel treatment were older, had a lower glomerular filtration rate, and had less frequently a positive history of acute coronary syndrome or of PCI with stent implantation before the last procedure.

**Table 1:** Demographic, clinical and procedural characteristics of the study patients according to the anti-P2Y12 drug at inclusion.

	Clopidogrel (*n* = 75)	Prasugrel (*n* = 24)	Ticagrelor (*n* = 30)	*p*
**Demographics**				
∙ Race (Caucasian)	74 (98.7)	24 (100.0)	30 (100.0)	0.701
∙ Age, years	65.3 ± 10.4	57.5 ± 10.8	59.4 ± 10.4	0.002
∙ Sex (Male)	62 (82.7)	21 (87.5)	27 (90.0)	0.602
∙ BMI, kg/m2	27.6 ± 3.5	29.0 ± 4.7	26.5 ± 2.9	0.048
**Cardiovascular risks factors**				
∙ Diabetes	15 (20)	5 (20.8)	6 (20.0)	0.996
∙ Hypertension	59 (78.7)	16 (66.7)	17 (56.7)	0.068
∙ Dyslipidemia	68 (90.7)	20 (83.3)	23 (76.7)	0.162
∙ Smoker (current or former)	49 (65.3)	18 (75.0)	21 (70.0)	0.662
**Indication to revascularization**				
∙ Stable angina	24 (32)	3 (12.5)	3 (10.0)	0.021
∙ Unstable angina	14 (18.7)	5 (20.8)	2 (6.7)	0.262
∙ Positive functional test	13 (17.3)	1 (4.2)	1 (3.3)	0.059
∙ Elective stent post ACS	30 (40)	15 (62.5)	24 (80.0)	0.001
∙ Drug eluted stents	1.4 ± 0.7	1.3 ± 0.5	1.7 ± 1.3	0.143
**Prior cardiac history**				
∙ ACS	39 (52.0)	18 (75.0)	24 (80.0)	0.010
∙ Coronary artery bypass graft	11 (14.7)	3 (12.5)	1 (3.3)	0.264
∙ PCI without stent	11 (14.7)	1 (4.2)	0 (0)	0.041
∙ PCI with stent	38 (50.7)	17 (70.8)	23 (76.7)	0.024

*Values are expressed as means ± SD or n with percentages between brackets. ACS, acute coronary syndrome; BMI, body mass index; eGFR ckd-epi, estimated glomerular function rate - chronic kidney disease epidemiology collaboration; PCI, percutaneous coronarography intervention.*

**Table 2:** Pharmacotherapy, biological results and genotyping of the study patients according to the anti-P2Y12 drug at inclusion.

	Clopidogrel (*n* = 75)	Prasugrel (*n* = 24)	Ticagrelor (*n* = 30)	*p*
**Pharmacotherapy**				
∙ Aspirin	74 (98.7)	24 (100.0)	30 (100.0)	0.701
∙ Beta-blocker	54 (72.0)	21 (87.5)	27 (90.0)	0.066
∙ RAAS -inhibitor	63 (84.0)	21 (87.5)	24 (80.0)	0.760
∙ Calcium channel blocker	7 (9.3)	1 (4.2)	4 (13.3)	0.521
∙ Diuretic	14 (18.7)	3 (12.5)	4 (13.3)	0.691
∙ Statin	71 (94.7)	23 (95.8)	29 (96.7)	0.903
**Laboratory results**				
∙ Serum creatinine, μmol/l	89.5 ± 20.8	89.1 ± 12.6	82.3 ± 8.5	0.159
∙ eGFR ckd-epi, ml/min/1.73 m^2^	77.0 ± 17.1	81.2 ± 16.7	86.9 ± 12.6	0.019
∙ Total cholesterol, mmol/l	4.2 ± 1.0	4.1 ± 0.7	4.0 ± 0.7	0.548
∙ LDL-cholesterol, mmol/l	2.3 ± 0.7	2.2 ± 0.5	2.0 ± 0.6	0.286
∙ HDL-cholesterol, mmol/l	1.3 ± 0.4	1.2 ± 0.3	1.2 ± 0.3	0.428
∙ Fasting glucose, mmol/l	6.2 ± 1.6	6.2 ± 1.1	5.8 ± 0.8	0.399
∙ Hemoglobin, g/l	143.1 ± 11.7	148.8 ± 11.6	148.3 ± 11.5	0.035
**Genotyping**				
∙ CYP2C19^∗^2 allele carriers	20 (26.7)	6 (25.0)	10 (33.3)	0.747

*Values are expressed as means ± SD or n with percentages between brackets. RAAS, renin-angiotensin-aldosterone-system.*

### VASP-PRI Results, HPR and Predictors of the On-treatment Anti-P2Y12 Drug Response

**FIGURE 2:**
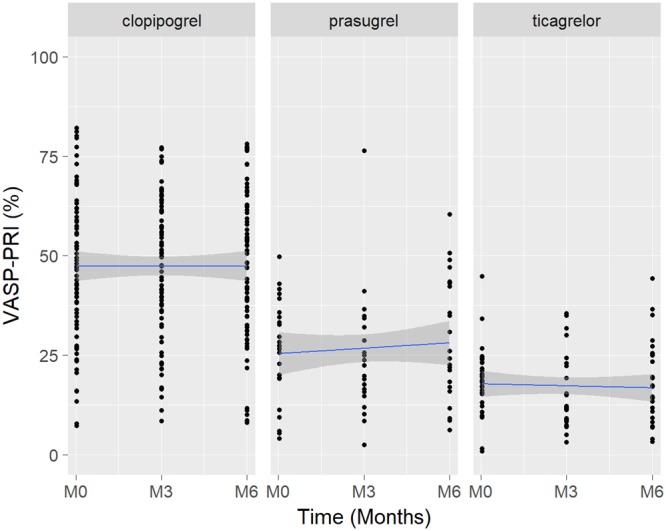
Boxplot of VASP-PRI results over time according to the anti-P2Y12 drug. VASP-PRI, vasodilator-stimulated-phosphoprotein-phosphorylation-platelet- reactivity-index.

VASP-PRI values at inclusion, 3 and 6 months for each anti-P2Y12 drug are shown in **Figure 2**.

VASP-PRI at inclusion and 6-month was lowest in ticagrelor treated patients. Mean ± SD VASP-PRI at inclusion was 18.6 ± 8.5% with ticagrelor, 26.4 ± 12.7% with prasugrel and 47.6 ± 18.4% with clopidogrel (ANOVA *p* < 0.0001). VASP-PRI at 6-month was 17.7 ± 11.0% with ticagrelor, 29.2 ± 15.5% with prasugrel and 47.2 ± 17.6 % with clopidogrel (*p* < 0.0001).

Six-months efficacy was superior both when comparing ticagrelor to clopidogrel (*p* < 0.0001) and ticagrelor to prasugrel (*p* = 0.0046). We observed large between-patient variability at each visit and in the three treatment groups. Nevertheless, overall mean VASP-PRI values remained stable over the 6-months period in the three groups (*p* = 0.691 for ticagrelor, *p* = 0.849 for clopidogrel, *p* = 0.525 for prasugrel). The proportion of HPR at inclusion was 0% with ticagrelor and prasugrel, vs.45.3% with clopidogrel (*p* < 0.0001); at 6 months, this proportion was 0% with ticagrelor, 12.5% with prasugrel and 49.3% with clopidogrel (*p* < 0.0001).

When applying linear mixed-effects models to model longitudinal VASP-PRI data, significant differences between treatment groups were confirmed (*P* ≤ 0.001 for absolute VASP-PRI values, *P* ≤ 0.001 for HPR). No significant effect of time was observed (*P* = 0.309 for absolute VASP-PRI values, *P* = 0.140 for HPR) at 6 months.

Anti-P2Y12 drugs (*p* ≤ 0.001) and diabetes (*p* = 0.014) emerged as predictors of poor antiplatelet response after adjusting for age, sex, BMI, and CYP2C19^∗^2 carriers status.

CYP2C19^∗^2 carriers status was associated with HPR only in patients treated with clopidogrel (*p* = 0.0083 with clopidogrel; *p* = 0.0678 with prasugrel; *p* = 0.2931 with ticagrelor).

### Results of Adherence Summary Statistics

**FIGURE 3:**
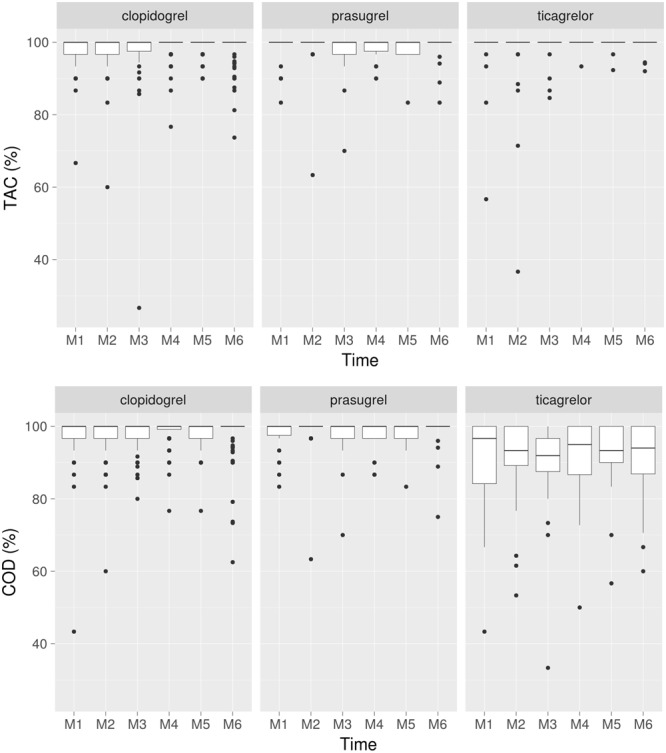
Taking adherence **(upper)** and correct adherence **(lower)** over the 6-months monitoring period according to the anti-P2Y12 drug. Correct adherence (*Cod*), proportion of days with correct dosing during the time window; Taking adherence (*Tac*), proportion of prescribed drug taken during the time window.

**Figure 3** shows the monthly adherence summary statistics over the 6-months of monitoring according to the P2Y12 receptor antagonist treatment group.

**FIGURE 4:**
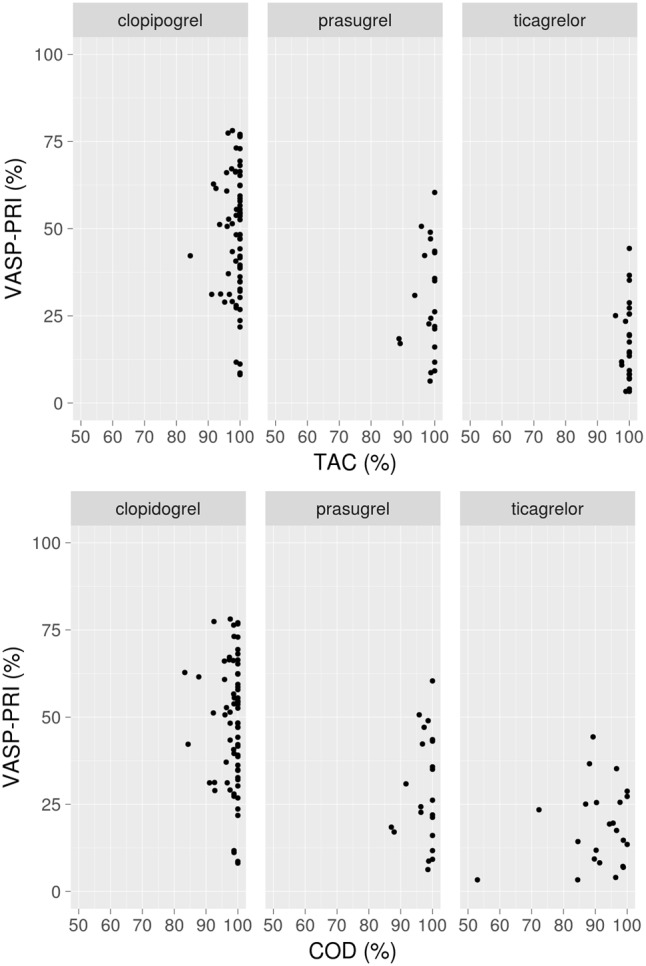
Association between VASP-PRI at 6-months and taking adherence **(upper)** and correct adherence **(lower)** in the 30 days preceding the VASP-PRI assay. VASP-PRI, vasodilator-stimulated-phosphoprotein-phosphorylation-platelet-reactivity-index.

Adherence statistics were lowest in ticagrelor vs. clopidogrel and prasugrel treated patients: median [IQR] *taking adherence (Tac)* was 96 [82–100]% with ticagrelor, 100 [99–101]% with clopidogrel and 100 [97–101]% with prasugrel (*p* = 0.0001); median [IQR] *correct dosing (Cod)* was 88 [73–95]% with ticagrelor, 98 [96–99]% with clopidogrel and 97 [92.5–98]% with prasugrel (*p* = 0.0001). **Figure 4** shows the 6-month VASP-PRI results in the three anti-P2Y12 drugs according to the *Tac* and *Cod* during the month before VASP-PRI assessment.

### Results of Safety

**Table 3** reports all adverse events occurring during the 6-months study period. Specifically, we did not observe a higher incidence of bleeding episodes under prasugrel and ticagrelor, despite their more powerful platelets inhibition.

**Table 3:** Adverse events at 6 months.

	Total (*n* = 129)	Clopidogrel (*n* = 75)	Prasugrel (*n* = 24)	Ticagrelor (*n* = 30)	*P*-value
**Ischemic events, *N* (%)**	4 (3.2)	2 (2.7)	1 (4.2)	1 (3.9)	0.598
• Unstable angina	3 (2.4)	2 (2.7)	1 (4.2)	0 (0)	0.462
• ACS	0 (0)	0 (0)	0 (0)	0 (0)	
• Stent thrombosis	1 (0.8)	0	0	1 (3.9)	0.152
**Coronary interventions, *N* (%)**	14 (10.9)	7 (9.3)	5 (21.7)	2 (7.7)	0.274
• Angioplasty	12 (9.7)	5 (6.7)	5 (21.7)	2 (7.7)	0.197
**Bleeding (GUSTO criteria), *N* (%)**	67 (65.9)	38 (50.7)	19 (79.2)	19 (93.3)	0.102
• Severe or Life-threating	0 (0)	0 (0)	0 (0)	0 (0)	
• Moderate	0 (0)	0 (0)	0 (0)	0 (0)	
• Mild	67 (65.9)	38 (50.7)	19 (79.2)	19 (93.3)	0.10

*Values are expressed as percentages between brackets. ACS, acute coronary syndrome; GUSTO (Global Utilization Of Streptokinase And Tpa For Occluded Arteries) definition for bleeding: Severe or Life-threatening (intracerebral hemorrhage or bleeding resulting in substantial hemodynamic compromise requiring treatment), Moderate (requiring blood transfusion but not resulting in hemodynamic compromise), Mild (bleeding that does not meet above criteria).*

Three patients had to stop the trial prematurely: one clopidogrel treated patient in the context of recurrent vesical bleeding, one ticagrelor treated patient had to been switched to an alternative PY12 receptor inhibitor because of nausea and headache, in a third case ticagrelor was stopped by the treating physician because of fear of interactions with other drugs.

We did not observe any between-treatment groups difference in the incidence of other adverse events (data not shown).

## Discussion

The main finding of this study is that despite a slightly lower adherence to ticagrelor, P2Y12 inhibition is strongest with this drug after 6 months of treatment compared to prasugrel and clopidogrel.

To our knowledge, this is the first study assessing the impact of long-term (6–12 months) adherence on P2Y12 receptor inhibition. The clinical impact of antiplatelet drugs discontinuation after PCI with DES implantation in the subacute phase (>1 month) after stent implantation is controversial. First generation DES has increased the risk for stent thrombosis beyond the traditional subacute (first month) period as compared with bare-metal stents (Daemen et al., 2007). Non-adherence to duals antiplatelet therapy (DAPT) in patients treated with DES, defined as missing ≥1 days of either medication within the first 6 months, has been independently associated with adverse thrombotic outcomes at 1 year. In contrast, non-adherence after 6 months has not been associated with increased subsequent risk for the composite endpoint of death or myocardial infarct, if the patient had been fully adherent to DAPT during the first 6 months (Cutlip et al., 2015).

These data are in contrast to several studies reporting no adverse impact of DAPT discontinuation after the first month (Ferreira-Gonzalez et al., 2012; Naidu et al., 2012; Silber et al., 2014).

Taking adherence results – the proportion of prescribed drug taken during the time window, reflecting both the average dose received over a given period and the total dose received over that period -, were excellent and stable over the 6-months monitored period in the three treatment groups; ticagrelor treated patients showed a slightly lower taking adherence.

When refining the analysis, the percentage of days with drug taken as prescribed was significantly lower in patients treated with ticagrelor b.i.d. compared to o.d.

When analyzing the drug dosing histories of 5014 patients extracted from an electronically compiled dosing history database, Vrijens et al. (2014) showed a higher likelihood of missing a dose in patients on b.i.d. ticagrelor regimen with respect to o.d. clopidogrel. Their main finding was that although patients treated twice daily more frequently missed single dose compared with patients treated once daily, the calculated level of platelet inhibition remained higher for ticagrelor twice daily compared with clopidogrel once daily. They underlined the need to shift focus from percentages of prescribed doses taken to pharmacometrically equivalent dosing errors, which should be better suited to predict therapeutic consequences of missed doses.

In analogy with the above mentioned study findings, we showed no meaningful pharmacodynamic impact of these execution irregularities on the ticagrelor-induced platelets inhibition.

**FIGURE 5:**
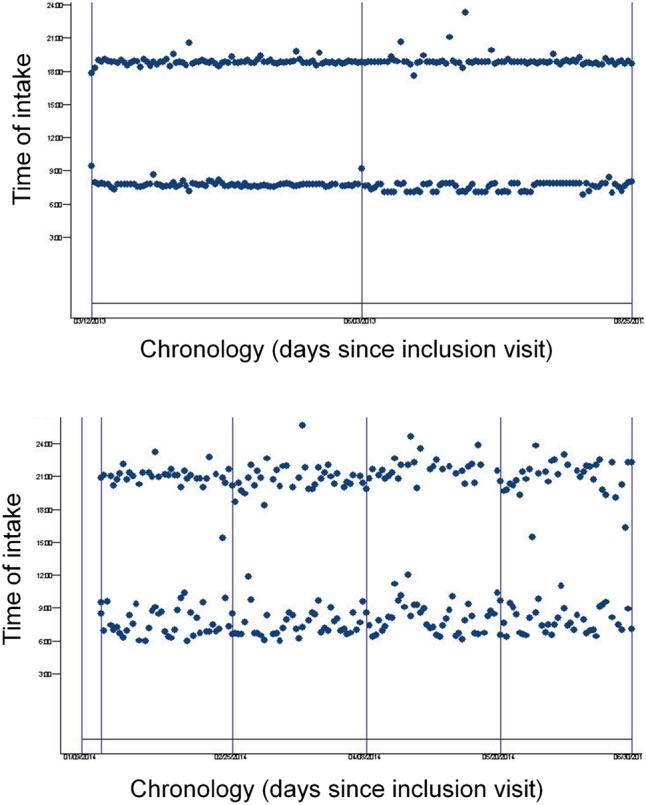
Dosing chronology plot used to visualize the dosing history of two patients on ticagrelor b.i.d [Patient 1 **(upper)**, Patient 2 **(lower)**]. Dosing chronology plot of two participants. The horizontal axis displays the days since the inclusion visit. The vertical axis shows the time of drug intake (24 h clock; dosing time). Dots represent opening of the electronic monitor (Medication Event Monitoring System-MEMS^®^).

The lack of therapeutic consequences of drug intake irregularities on medicament efficacy is highlighted by **Figure 5**, which show the dosing history of two patients on ticagrelor b.i.d. The electronically compiled drug dosing history data reveal major differences in the dynamics over time. Patient 1 (upper panel) executed the dosing regimen perfectly (taking adherence 100%, correct dosing 100%). Patient 2 (lower panel) was persistent throughout the observation period, but showed several irregularities in the drug intake (taking adherence 94.1%, correct dosing 70.6%). Despite the different execution profile, the ticagrelor-induced antiplatelet response was only slightly better in patient 2 (6-months VASP-PRI 28.8% in patient 1, 23.4% in patient 2).

### Limitations of the Study

The baseline imbalance in age and cardiovascular antecedents (the clopidogrel population was older but had less severe cardiovascular history) may have introduced a bias, increasing the between-treatment groups VASP-PRI differences. These differences reflect the fact that the anti-P2Y12 drug choice was not randomized, but left to the choice of the cardiologists. Prasugrel has shown no net superiority with respect to clopidogrel in very elderly patients (≥75 years) and in those weighing less than 60 kg due to an increase in bleeding complications (Wiviott et al., 2007). Ticagrelor has shown a net benefit when compared to clopidogrel in patients after acute coronary syndrome. Prasugrel and ticagrelor are now indicated in patients resistant or poorly responsive to clopidogrel with prior recurrent cardiovascular events (Wallentin et al., 2009).

The high adherence observed in this study does not necessarily reflect the real-life situation. In the trial, all participants were aware that their adherence was being monitored and had regular appointments to attend, during which the adherence results were discussed. This approach has been proven to enhance successfully adherence to medications (Demonceau et al., 2013). As adherence was high in all groups, it cannot be excluded that an effect on platelet inhibition would have been seen under a specific cutoff level of adherence.

This study explored only a subset of factors potentially implicated in the long-term P2Y12 receptor inhibition. Clinical, demographic, genomic factors not yet identified will probably emerge in the future and will further clarify these complex interactions. Procedural heterogeneity in sampling treatment could have influenced VASP-PRI results, but the risk was minimized by a strict standardized protocol for drug intake and blood sampling and by centralizing the analysis in one laboratory.

## Conclusion

Drug adherence to anti-P2Y12 drugs assessed with electronic monitoring was very high in patients after elective PCI. When adherence is high, the newer anti-P2Y12 drugs ticagrelor and prasugrel showed higher antiplatelet inhibition and less response variability with respect to other anti-P2Y12 drugs.

These data suggest that when adherence to oral anti-P2Y12 drugs is high, the pharmacokinetic-pharmacodynamic differences are more important than adherence in determining platelets’ responsiveness.

## Author Contributions

The authors’ responsibilities were as follows: GW, VF, and MB: wrote the manuscript; GW, MB, VF, and ET: designed research; GW, VF, IM, IB, MB, CE, and PF: performed research; BV, ET, GW, IB, OM, and VF: analyzed data. All authors critically revised the manuscript for important intellectual content and approved the final version of the manuscript.

## Conflict of Interest Statement

MB declares to have received research grants from the Swiss federal office of technology and professional training, from the Swiss Heart, from Astra-Zeneca (Switzerland) and Sanofi (Switzerland). However these societies were not involved in the analysis of data and redaction of the paper. The other authors declare that the research was conducted in the absence of any commercial or financial relationships that could be construed as a potential conflict of interest.
