# Supplementary material for: Comparative Long-Term Effect of Three Anti-P2Y12 Drugs after Percutaneous Angioplasty: An Observational Study Based on Electronic Drug Adherence Monitoring
Source: Front Pharmacol. 2017 Oct 25;8:738. doi: 10.3389/fphar.2017.00738 (PMC5660969; doi:10.3389/fphar.2017.00738)
Supplement: Supplementary file 1 [file Table_1.PDF]

frontiers

Supplementary Material

Comparative long-term effect of three anti-P2Y12 drugs after percutaneous angioplasty:

an observational study based on electronic drug adherence monitoring

Valentina Forni Ogna[1*]; Isabelle Bassi[1]; Isabelle Menetrey[1]; Olivier Muller[2]; Eric Tousset[3]; Pierre Fontana[4]; Eric Eeckhout[2]; Chin B. Eap[5, 6]; Bernard Vrijens[3]; Michel Burnier[1] and Gregoire Wuerzner[1]

*** Correspondence**: Valentina Forni Ogna: valentina.forni@chuv.ch

Table 1: Demographic, clinical and procedural characteristics of included (adherence group) versus not included (no-adherence group).

	**Not included (*n*=62)**	**Included (*n*=129)**	**p**
**Demographics**			
• Race (Caucasian)	60 (96.8)	128 (99.2)	0.097
• Age, yrs	63 ± 10.1	62.5 ± 10.9	0.767
• Sex (Male)	55 (88.7)	110 (85.3)	0.421
• BMI, kg/m2	27.8 ± 3.5	27.6 ± 3.7	0.789
**Cardiovascular risks factors**			
• Diabetes	19 (30.6)	26 (20.1)	0.110
• Hypertension	47 (75.8)	92 (71.3)	0.426
• Dyslipidemia	54 (87.1)	111 (86)	0.393
• Smoker (current or former)	45 (72.6)	88 (68.2)	0.377
**Indication to revascularization**			
• Stable angina	22 (35.5)	30 (23.3)	0.075
• Unstable angina	6 (9.7)	21 (16.3)	0.220
• Positive functional test	17 (27.4)	15 (11.6)	0.006
• Elective stent post ACS	22 (35.5)	69 (53.5)	0.020
• Drug eluted stents	1.5 ± 0.6	1.4 ± 0.8	0.365

**Prior cardiac history**			
• ACS	49 (79.0)	105 (81,4)	0.695
• Coronary artery bypass graft	34 (54.8)	81 (62.8)	0.293
• PCI without stent	9 (14.5)	15 (11.6)	0.573
• PCI with stent	12 (19.3)	12 (9.3)	0.050

Values are expressed as means±SD or n with percentages between brackets.

ACS=acute coronary syndrome; BMI=body mass index; eGFR ckd-epi=estimated glomerular function rate - chronic kidney disease epidemiology collaboration; PCI=percutaneous coronarography intervention.
